# Serum Rcn3 level is a potential diagnostic biomarker for connective tissue disease-associated interstitial lung disease and reflects the severity of pulmonary function

**DOI:** 10.1186/s12890-023-02360-4

**Published:** 2023-02-19

**Authors:** Fangping Ding, Liu Yang, Yingfei Wang, Jing Wang, Yingmin Ma, Jiawei Jin

**Affiliations:** 1 Department of Respiratory and Critical Care Medicine, Beijing Chaoyang Hospital, Capital Medical University, N0.5 Jingyuan Road, Beijing, 100043 China; 2 Department of Respiratory and Critical Care Medicine, Beijing Youan Hospital, Capital Medical University, No.8 Xi Tou Tiao, Youanmen Wai, Beijing, 100069 China; 3 The Clinical Research Center, Beijing Chaoyang Hospital, Capital Medical University, Beijing, 100043 China; 4 Beijing Institute of Respiratory Medicine, Capital Medical University, Beijing, 100020 China

**Keywords:** Reticulocalbin 3, Connective tissue disease-associated interstitial lung disease, Idiopathic pulmonary fibrosis, Biomarker

## Abstract

**Background:**

Although reticulocalbin 3 (Rcn3) has a critical role in alveolar epithelial function as well as in pathogenesis of pulmonary fibrosis, no study has yet examined its diagnostic and prognostic values for interstitial lung disease (ILD). This study aimed to evaluate Rcn3 as a potential marker in differential diagnosis of idiopathic pulmonary fibrosis (IPF) and connective tissue disease-associated interstitial lung disease (CTD-ILD) and in reflecting the severity of disease.

**Methods:**

This was a retrospective observational pilot study included 71 ILD patients and 39 healthy controls. These patients were stratified into IPF group (39) and CTD-ILD group (32). The severity of ILD was evaluated through pulmonary function test.

**Results:**

Serum Rcn3 level was statistically higher in CTD-ILD patients than that in IPF patients (*p* = 0.017) and healthy controls (*p* = 0.010). Serum Rcn3 further showed statistically negative correlation with pulmonary function indexes (TLC% pred and DLCO% pred) and positive correlation with inflammatory indexes (CRP and ESR) (r =  − 0.367, *p* = 0.039; r =  − 0.370, *p* = 0.037; r = 0.355, *p* = 0.046; r = 0.392, *p* = 0.026, respectively) in CTD-ILD patients rather than IPF patients. ROC analysis demonstrated that serum Rcn3 had superior diagnostic value for CTD-ILD and a cutoff value of 2.73 ng/mL had a sensitivity of 69%, a specificity of 69% and an accuracy of 45% for diagnose of CTD-ILD.

**Conclusions:**

Serum Rcn3 levels might be a clinically useful biomarker in screening and evaluating CTD-ILD.

## Introduction

Interstitial lung disease (ILD) is characterized as a group of diffuse parenchymal lung disorders and its heterogeneous pathogenesis leads to high morbidity and mortality [1]. The earlier diagnosis and discrimination are thus important for improving its treatment and prognosis. Idiopathic pulmonary fibrosis (IPF) and connective tissue disease-associated interstitial lung disease (CTD-ILD) are the two most common subtypes of ILD, in which CTD-ILD has a relatively less poor prognosis versus IPF [2, 3]. Although the IPF and CTD-ILD patients (especially in patients with rheumatoid arthritis) can show similar usual interstitial pneumonia (UIP) pattern in a chest CT examination, their pathogenetic mechanisms and therapeutic strategies are distinct [4–6]. Therefore, an effective differential diagnosis between them is essential for clinicians.

The poor prognosis of ILD is associated with obvious clinical manifestations including the dyspnea score, declined pulmonary function degree and progressive CT patterns. These clinical changes represent the progression and severity of ILD, but they are not convenient for both patients and clinicians in prediction of ILD prognosis. Recently, increasing studies showed some biomarkers along with CT images to predict the severity and progression of ILD. The decrease of Vitamin D level and the increase of serum KL-6 level in patients with CTD-ILD were correlated with ILD severity and poor prognosis [7, 8]. Additionally, serum surfactant protein-D (SP-D) level was associated with the decline of forced vital capacity (FVC) in CTD-ILD patients [9]. Nevertheless, none of these biomarkers have been used in clinical practice and in the differential diagnosis between CTD-ILD and IPF.

Reticulocalbin 3 (Rcn3) is an endoplasmic reticulum lumen protein localized to the secretory pathway [10]. Our previous studies indicated an indispensable physiological role of Rcn3 in alveolar epithelium during lung development and lung injury-repair in adult lung [11, 12]. The Rcn3 expression in alveolar epithelium is markedly enhanced in bleomycin-induced pulmonary fibrosis, while selective deletion of Rcn3 in type II alveolar epithelial cells (AECIIs) exacerbated bleomycin-induced lung fibrosis, suggesting a protective role of Rcn3 in lung fibrosis [12]. In addition, Rcn3 was also identified as a fibroblast-specific biomarker of poorer prognosis of colorectal cancer (CRC) in a recent study [13]. These findings suggest the importance of Rcn3 in pulmonary interstitial remodeling. However, to date, no studies have investigated the clinical value of Rcn3 in the diagnosis of ILD as well as the correlation of Rcn3 expression with ILD severity.

Given the distinct pathologists between IPF and CTD-ILD, we hypothesis that Rcn3 level may show different diagnostic value in these two groups. To test our hypothesis, we performed a retrospective observational pilot study on the existing ILD patient serum samples to compare the serum Rcn3 level in these two types of ILD patients and further examined the statistic correlations of Rcn3 level to severity in these patients. The value of Rcn3 level for differential diagnosis of them was also examined. Our findings could shed light on developing earlier biomarker for the progression of ILD.

## Methods

### Study design

This was a retrospective pilot study on existing ILD data (serum samples were collected between January 2020 and December 2021 in the department of respiratory and critical care medicine, Beijing Chao-Yang hospital), which was approved by the Ethics Committee of Beijing Chao-Yang Hospital (2021-KE-295). All methods were carried out in accordance with relevant guidelines and regulations and the written informed consent was obtained from all subjects. A total of 71 patients with ILD presenting UIP or probable UIP patterns in chest CT examination were included in the study. IPF and CTD-ILD patients were defined according to the published criteria of the corresponding societies [14–19]. Patients with acute infection or cancer, and who had corticosteroids using history or incomplete data were excluded. The healthy controls were volunteers with a normal chest CT examination from the physical examination center of the hospital during the same period approved by 2021-KE-295 and 2021-KE-313.

### Data collection

The patients with the initial diagnosis of CTD-ILD and IPF were enrolled and their serum samples were collected at admission, the pulmonary function data were collected within 2 days after admission. The clinical data included gender, age, smoking history, underlying diseases, vital signs, laboratory data and pulmonary function test were obtained from medical records. Rcn3 concentration (ng/mL) was measured through Elisa kits from Cloud-clone.

### Statistical analysis

Normally distributed continuous variables were presented as means ± standard deviations (SD) and statistical comparison between two groups were performed by two-tailed student’s t-test. The non-normally distributed continuous variables were presented as median and interquartile range (IQR, 25th–75th percentiles) and the Mann–Whitney U test was used for the comparison between the two groups. In adjusting comparison, gender and smoking index were adjusted using logistic regression model and general linear model, respectively. The statistical comparison of three groups were performed using one-way ANOVA followed by post hoc multiple comparison tests where appropriate. Categorical variables were depicted as numbers and percentages and analyzed with chi-squared test or Fisher’s exact test. Pearson correlation analysis was used to evaluate the correlation between Rcn3 and pulmonary function indexes [predicted total lung capacity (TLC% pred), predicted forced vital capacity (FVC % pred) and predicted lung diffusing capacity for carbon monoxide (DLCO% pred)] and inflammatory indexes [C-reactive protein (CRP) and erythrocyte sedimentation rate (ESR)]. Multivariate linear regression was further used to determine the independent association of Rcn3 and pulmonary function indexes according to CRP and ESR.

Receiver operating characteristic (ROC) curve was depicted, and the area under the curve [AUC, with 95% confidence interval (CI)] was calculated to evaluate the value of serum Rcn3 level for the diagnosis of CTD-ILD from IPF. Furthermore, the sensitivity, specificity, predictive values, likelihood ratios and the diagnostic accuracy of the suggested cut-off Rcn3 value were calculated. A two-sided *p* value of < 0.05 was considered statistically significant, and all analyses were performed with SPSS (version 19.0, SPSS, Chicago, IL, USA).

## Results

### The serum Rcn3 level was significantly increased in CTD-ILD patients versus IPF patients

**Fig. 1:**
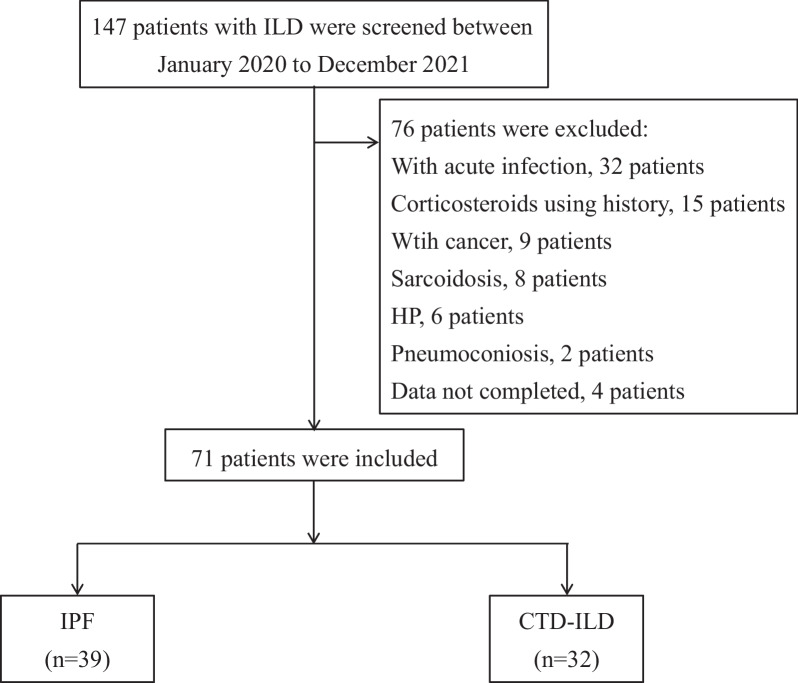
Flowchart of patient enrollment. ILD, interstitial lung disease; HP, hypersensitivity pneumonitis; IPF, idiopathic pulmonary fibrosis; CTD-ILD, connective tissue disease-associated interstitial lung disease

A total of 110 subjects were enrolled in the study, including 39 healthy controls and 71 patients diagnosed as ILD (Fig. 1, a flowchart of patient enrollment). The baseline clinical data of healthy people and patients at admission are shown in Table 1. There was no significant difference in sex, age, white blood cell, neutrophil granulocyte, lymphocyte, haemoglobin, platelet and serum Rcn3 level between the two groups, and Cohen’s d = 0.41 for the difference of serum Rcn3 between ILD patients and healthy controls.

**Table 1:** Characteristics of Healthy Controls or Patients with ILD

Variables	Healthy controls (n = 39)	ILD patients (n = 71)	*P*
Male, n (%)	18 (46.2)	37 (52.1)	0.550
Age (years)	61 ± 8	64 ± 10	0.120
Current smoker, n (%)	N/A	13 (18.6)	
Smoking index	N/A	0 (0 ~ 450)	
White blood cell (× 10^9^/L)	6.1 (5.5 ~ 6.8)	6.8 (5.8 ~ 8.1)	0.064
Neutrophil granulocyte (× 10^9^/L)	3.8 (3.0 ~ 4.1)	3.9 (3.1 ~ 5.0)	0.145
Lymphocyte (× 10^9^/L)	2.0 (1.7 ~ 2.3)	1.8 (1.3 ~ 2.3)	0.069
Haemoglobin (g/L)	134.2 ± 10.2	133.2 ± 16.2	0.712
Platelet (× 10^9^/L)	225 ± 44	217 ± 53	0.400
Rcn3 level in serum (ng/ml)	2.52 (1.88 ~ 3.19)	2.71 (2.02 ~ 4.17)	0.087

*ILD* Interstitial lung disease, *N/A* Not applicable

**Fig. 2:**
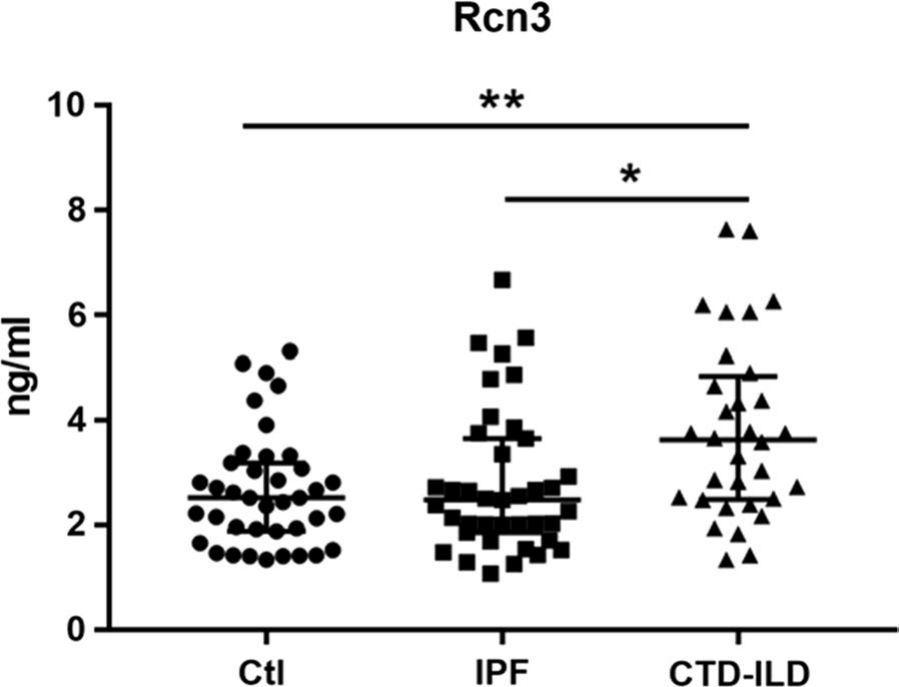
Serum Rcn3 level in healthy controls, patients with IPF or CTD-ILD. Ctl, healthy controls; IPF, idiopathic pulmonary fibrosis; CTD-ILD, connective tissue disease-associated interstitial lung disease; ns, no significance; *p < 0.05; **p < 0.01

We further stratified these patients into two groups: IPF group (n = 39) and CTD-ILD group (n = 32). As shown in Fig. 2, there was no significant difference in serum Rcn3 level between IPF patients and healthy controls, whereas patients with CTD-ILD exhibited significantly higher level of serum Rcn3 than controls (*P* = 0.010). Consistently, there was also a statistically significant difference in serum Rcn3 level between CTD-ILD and IPF patients (*P* = 0.017). These findings indicated a significant increase of serum Rcn3 level in patients with CTD-ILD, rather than IPF.

### The correlation of serum Rcn3 level with pulmonary function and inflammatory indexes in CTD-ILD and IPF patients

As shown in Table 2, the involved 32 CTD-ILD patients were diagnosed with Rheumatoid arthritis (n = 10), Sjogren's syndrome (n = 9), Sjogren's syndrome (n = 5), Dermatomyositis (n = 2), Systemic Sclerosis (n = 2) and Mixed connective tissue disease (n = 4). There was a statistically significant gender difference between patients with CTD-ILD and IPF (*P* = 0.026). In addition, the levels of CRP (*P* = 0.044), ESR (P = 0.026) and serum Rcn3 (*p* = 0.006) in CTD-ILD group were significantly higher than those in IPF group, Cohen’s d = 0.631 for the difference of serum Rcn3 between IPF and CTD-ILD patients. Furthermore, the serum Rcn3 level consistently showed significant difference between the two groups after gender-adjusted (*p* = 0.033) or smoking index-adjusted (*p* = 0.009). However, there were no significant differences in pulmonary function indexes (TLC% pred, FVC% pred and DLCO% pred) between CTD-ILD and IPF patients.

**Table 2:** Characteristics of patients with IPF or CTD-ILD

Variables	IPF (n = 39)	CTD-ILD (n = 32)	*P*
Male, n (%)	25 (64.1)	12 (37.5)	0.026*
Age (years)	66 ± 9	62 ± 11	0.142
Current smoker, n (%)	8 (20.5)	5 (15.6)	0.596
Smoking index	60 (0 ~ 450)	0 (0 ~ 438)	0.382
*Underlying diseases, n (%)*			
Hypertension	18 (46.2)	8 (25.0)	0.066
Diabetes	15 (38.5)	7 (21.9)	0.133
Cardiovascular disease	13 (33.3)	8 (25.0)	0.444
Cerebrovascular disease	4 (10.3)	2 (6.3)	0.683
Subtypes of CTD, n (%)			
Rheumatoid arthritis	N/A	10 (31.2)	
Sjogren's syndrome	N/A	9 (28.1)	
Systemic lupus erythematosus	N/A	5 (15.6)	
Dermatomyositis	N/A	2 (6.3)	
Systemic Sclerosis	N/A	2 (6.3)	
Mixed connective tissue disease	N/A	4 (12.5)	
*Radiologic patterns of ILD, n(%)*			0.168
UIP	36 (92.3)	25 (78.1)	
Probable UIP	3 (7.7)	7 (21.9)	
Onset of symptoms to hospital admission (days)	60 (30 ~ 120)	63 (19 ~ 321)	0.532
*Laboratory data at admission*			
Lactate (mmol/L)	1.20 (0.90 ~ 1.50)	0.95 (0.80 ~ 1.35)	0.134
White blood cell(× 10^9^/L)	6.8 (6.0 ~ 8.0)	6.9 (5.3 ~ 9.5)	0.644
Neutrophil granulocyte (× 10^9^/L)	3.8 (3.2 ~ 4.8)	4.2 (3.0 ~ 6.5)	0.329
Lymphocyte (× 10^9^/L)	1.9 (1.4 ~ 2.4)	1.6 (1.3 ~ 2.2)	0.186
Haemoglobin (g/L)	135.3 ± 14.7	130.5 ± 17.7	0.216
Platelet (× 10^9^/L)	208 ± 43	227 ± 62	0.141
C-reactive protein (mg/L)	5.0 (1.0 ~ 8.0)	6.0 (5.0 ~ 14.9)	0.044*
Procalcitonin (ng/ml)	0.05 (0.05 ~ 0.05)	0.05 (0.05 ~ 0.05)	0.703
ESR (mm/h)	15.0 (6.0 ~ 35.0)	32.5 (10.0 ~ 50.0)	0.024*
ALT (U/L)	17.6 (13.0 ~ 23.6)	16.2 (10.6 ~ 20.0)	0.119
AST (U/L)	20.2 (17.0 ~ 26.0)	19.9 (16.5 ~ 23.9)	0.488
Creatinine (umol/L)	72.2 ± 14.0	67.0 ± 12.1	0.099
D-Dimer (mg/L)	0.11 (0.07 ~ 0.23)	0.16 (0.11 ~ 0.39)	0.074
*Pulmonary function test*			
TLC% pred	76.0 (67.2 ~ 82.0)	75.4 (63.8 ~ 80.0)	0.627
FVC% pred	72.1 ± 10.7	72.5 ± 11.2	0.868
DLCO% pred	67.0 ± 12.3	63.2 ± 10.8	0.176
Rcn3 level in serum (ng/ml)	2.48 (1.85 ~ 3.65)	3.63 (2.49 ~ 4.84)	0.006*

*IPF* Idiopathic pulmonary fibrosis, *CTD-ILD* Connective tissue disease-associated interstitial lung disease, *UIP* Usual interstitial pneumonia, *ESR* Erythrocyte sedimentation rate, *AST* Aspartate transaminase, *ALT* Alanine aminotransferase, *TLC% pred* Predicted total lung capacity, *FVC% pred* Predicted forced vital capacity, *DLCO% pred* Predicted lung diffusing capacity for carbon monoxide, * *p* < 0.05

**Fig. 3:**
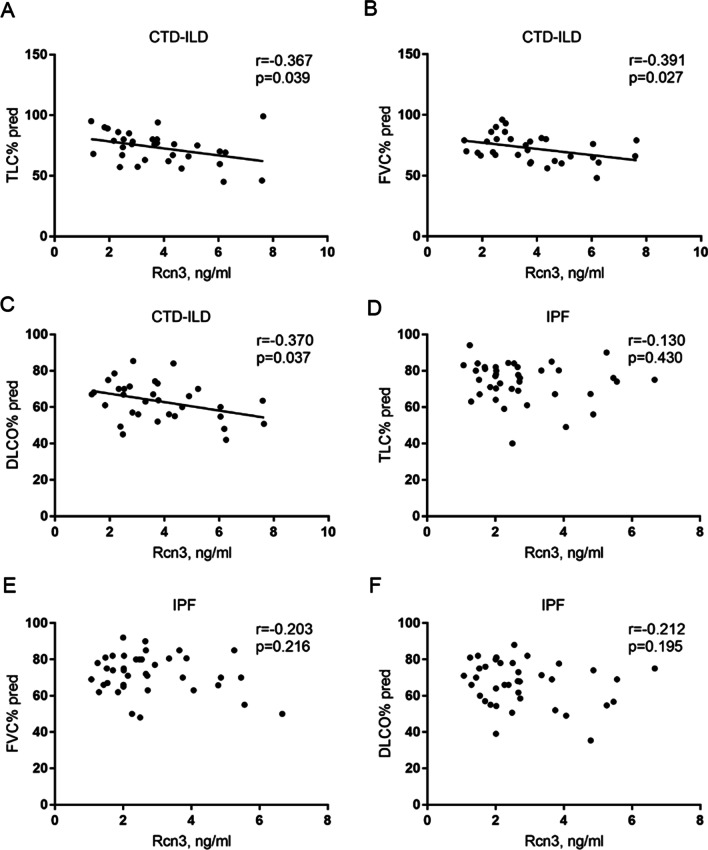
Scatter diagram for the relationship between serum Rcn3 level and pulmonary function indexes (TLC% pred, FVC% pred and DLCO% pred) in CTD-ILD patients (**A**–**C**) and IPF patients (**D**–**F**). CTD-ILD, connective tissue disease-associated interstitial lung disease; IPF, idiopathic pulmonary fibrosis; TLC% pred, predicted total lung capacity; FVC% pred, predicted forced vital capacity; DLCO% pred, predicted lung diffusing capacity for carbon monoxide

**Fig. 4:**
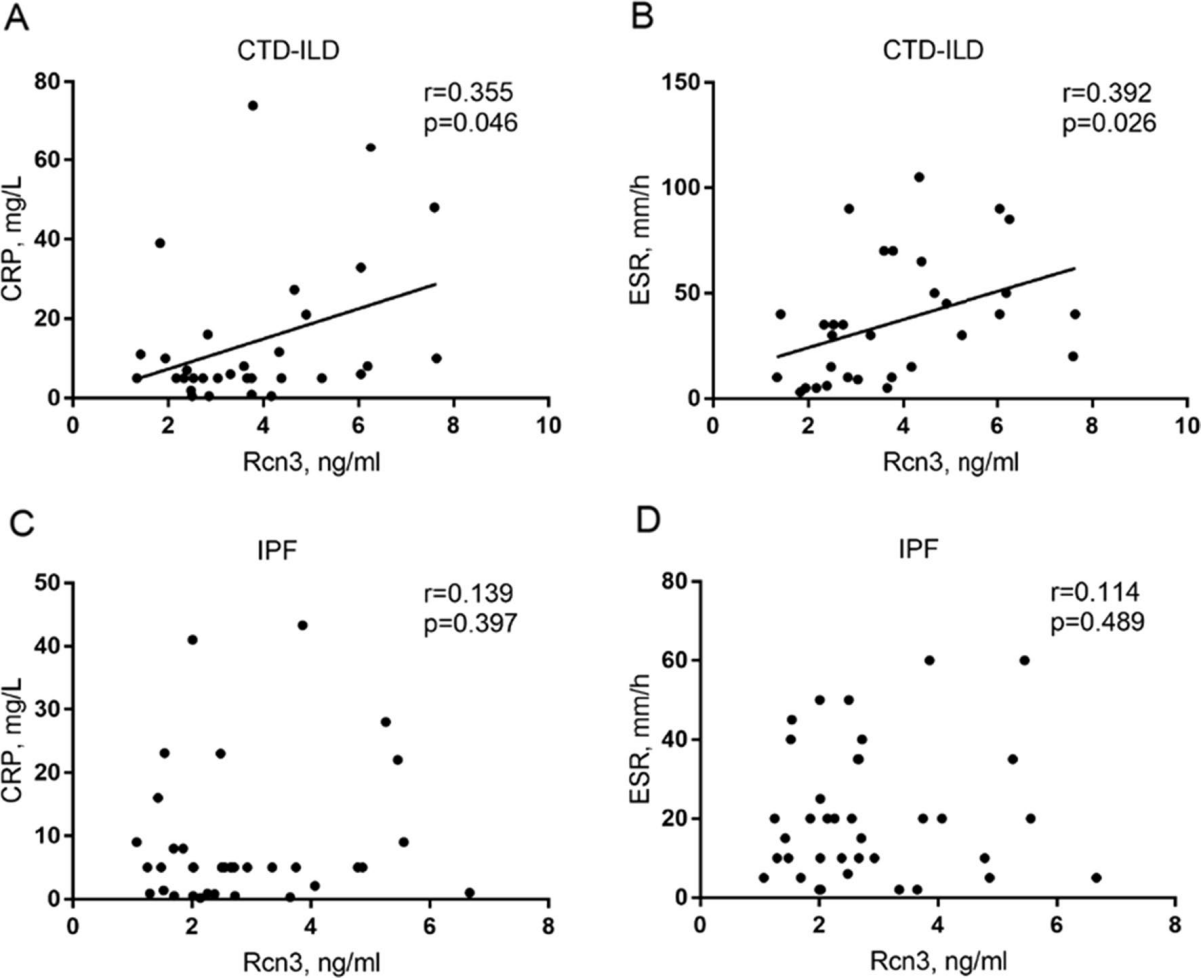
Scatter diagram for the relationship between serum Rcn3 level and inflammatory markers (CRP and ESR) in CTD-ILD patients (**A** and **B**) and IPF patients (**C** and **D**). CTD-ILD, connective tissue disease-associated interstitial lung disease; IPF, idiopathic pulmonary fibrosis; CRP, C-reactive protein; ESR, erythrocyte sedimentation rate

Of note, there were statistically negative correlations of serum Rcn3 level with TLC% pred, FVC% pred and DLCO% pred in CTD-ILD patients (r =  − 0.367, *p* = 0.039; r =  − 0.391, *p* = 0.027; r =  − 0.370, *p* = 0.037, respectively), whereases there were statistically positive correlations of serum Rcn3 level with CRP and ESR in patients with CTD-ILD (r = 0.355, *p* = 0.046; r = 0.392, *p* = 0.026, respectively). After adjustment for CRP and ESR levels, serum Rcn3 level in CTD-ILD patients consistently showed negative correlations with pulmonary function indexes TLC% pred (β =  − 0.447; 95% confidence interval =  − 6.639,  − 0.381; *p* = 0.029), DLCO% pred (β =  − 0.420; 95% confidence interval =  − 5.030,  − 0.247; *p* = 0.032) and FVC% pred (β =  − 0.360; 95% confidence interval =  − 4.834, 0.121; *p* = 0.061). However, significant correlations of serum Rcn3 level with these pulmonary function and inflammatory indexes were not observed in the IPF group (Figs. 3 and 4).

### The diagnostic ability of serum Rcn3 level for CTD-ILD

**Fig. 5:**
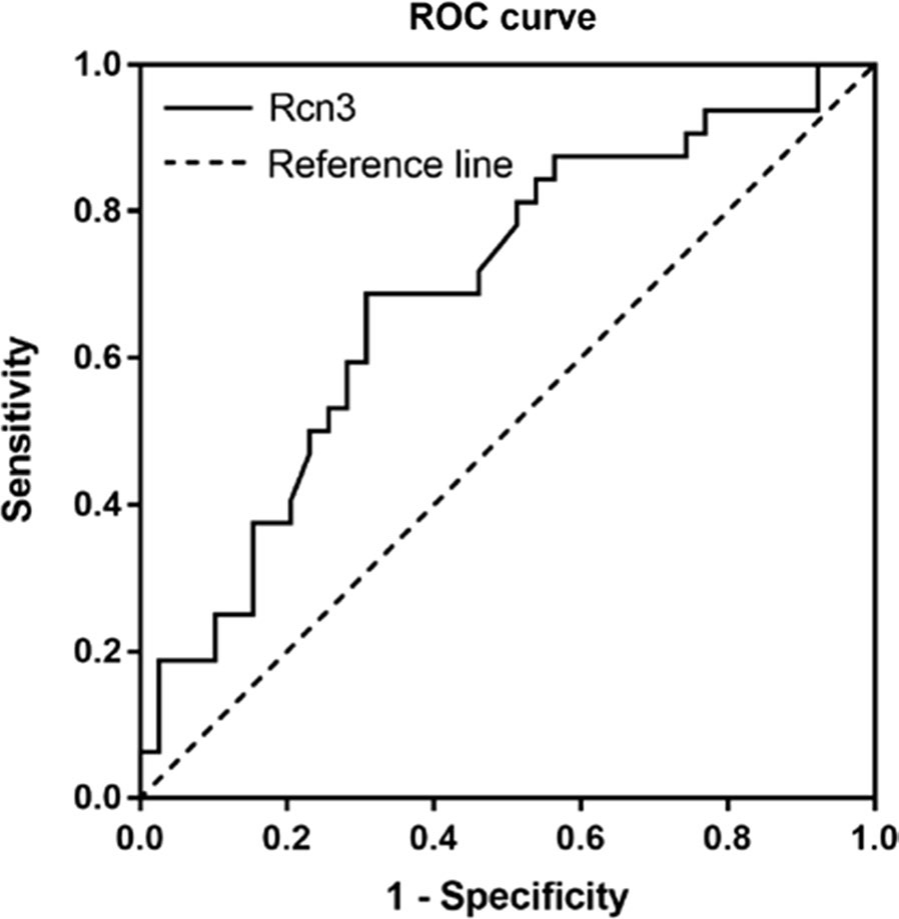
ROC curve for serum Rcn3 level in diagnosis of the CTD-ILD from IPF. ROC, receiver operating characteristic; CTD-ILD, connective tissue disease-associated interstitial lung disease; IPF, idiopathic pulmonary fibrosis

ROC analysis was performed on serum Rcn3 level to assess the sensitivity/specificity diagnostic value for CTD-ILD separated from IPF patients (Fig. 5). As shown in Table 3, the area under the ROC curve was 0.69 (*P* = 0.006, 95% CI 0.57–0.81). An Rcn3 cutoff value of 2.73 ng/mL had a sensitivity of 69%, a specificity of 69% and an accuracy of 45% for diagnose of CTD-ILD.

**Table 3:** Receiver operating characteristic curves data

Variables	Cutoff value	Sensitivity (%)	Specificity (%)	PPV (%)	NPV (%)	Accuracy (%)	LRPT	LRNT	Youden's index	AUC	*P* value
Rcn3 (ng/mL)	2.73	69	69	65	73	45	2.23	0.45	0.38	0.69	0.006
95% CI		0.50–0.83	0.52–0.82	0.46–0.80	0.56–0.86	0.33–0.57	1.32–3.78	0.26–0.77		0.57–0.81	

*PPV* Postive predictive value, *NPV* Negative predictive value, *LRPT* Likelihood ratio of positive test, *LRNT* Likelihood ratio of negative test, *AUC* Area under the curve, *CI* Confidence interval

## Discussion

This study, for the first time, investigated the clinical value of Rcn3 in ILD patients. The primary findings are as follows: the serum Rcn3 level in CTD-ILD patients was significantly higher than that in IPF patients; the serum Rcn3 level could be used as a biomarker to diagnose CTD-ILD from IPF; the severity of CTD-ILD represented by pulmonary function had a significantly positive correlation with serum Rcn3 level.

CTD-ILD is associated with significant morbidity and mortality due to sophisticated pathogenesis and limited treatment strategies, thus the earlier diagnosis and effective evaluation could be critical to improve the clinical outcome [20]. The diagnosis and prognostic prediction of CTD-ILD mainly depend on the clinical manifestations including the dyspnea score, pulmonary function degree and CT patterns, which are somewhat inconvenient for clinicians’ earlier decision making [21]. Therefore, the recent studies focused on looking for simple and effective biomarkers for diagnosis or to reflect the severity of ILD, such as Vitamin D, KL-6 and SP-D, although none of these biomarkers have been widely used in clinical practice [7]. Herein, we also indicated that the serum Rcn3 level in CTD-ILD patients is significantly higher than that in IPF patients and is significantly related with deleterious pulmonary function in CTD-ILD patients. Our data further suggested the diagnostic value of serum Rcn3 level: a cutoff of 2.73 ng/mL has a sensitivity of 69% and a specificity of 69% to diagnose CTD-ILD from IPF. The relatively low accuracy was likely due to the limited number of patients.

Both immune-inflammatory disorder and fibrogenesis are primary pathogenic mechanism of CTD-ILD, while the fibrogenesis caused by injury-repair deregulation primarily accounts for the pathogenesis of IPF. Our previous study indicates the functions of Rcn3 in alveolar epithelium including the pro-inflammatory function during acute lung injury and the anti-fibrotic function during injury-repair process [12, 22]. Consistently, there was also a statistically significant difference in serum Rcn3 level between CTD-ILD and IPF patients, which is likely due to the different contributions of Rcn3 in these two diseases. In addition, CTD-ILD patients, but not IPF patients, exhibited significantly higher level of serum Rcn3 than controls, suggesting that Rcn3 could be more sensitive to immune-inflammatory interstitial changes.

In addition to the higher serum Rcn3 levels in CTD-ILD patients than IPF patients, our study also showed a significantly positive correlation between serum Rcn3 level and the severity of CTD-ILD represented by pulmonary function indexes. We further demonstrated serum Rcn3 level in CTD-ILD patients was positively correlated with CRP and ESR. This finding suggests that Rcn3 could be more sensitive to immune-inflammatory deregulation-induced pulmonary interstitial remodeling, which could be explained by its critical role in fibrogenesis indicated by our previous studies on animal pulmonary fibrosis models [12]. Such hypothesis is in line with recent findings by other groups: Ding et al. [23] reported that Rcn3 showed a high expression in immune cells and fibroblast cells in Pan-cancer analysis; Park et al. [24] showed that Rcn3 was a pivotal regulator of collagen fibrillogenic during postnatal tendon development; Liu et al. [25] observed that Rcn3 was significantly up-regulated in keloids, in which abundant fibroblasts and collagen deposits involved. Together with these findings, our results showed that that serum Rcn3 levels could be sensitive and effective in predicting the severity of CTD-ILD due to the involvements of Rcn3 both in immune-inflammatory response and fibrogenesis, both of which are primary pathogenic mechanism of CTD-ILD. However, a perspective study enrolling more subjects is needed to validate the clinical value of Rcn3 in ILD.

There were some limitations of our present poilt study: (1) This was a retrospective study that included data from a single-center cohort and enrolled a small number of patients. (2) We missed a control group with CTD without ILD. (3) The limited sample size would probably not allow for an accurately estimating whether smoking would have effect on serum Rcn3 level as well as whether the serum Rcn3 levels are different according to the types of CTD such as rheumatoid arthritis, systemic sclerosis, and inflammatory myositis. (4) All subjects we enrolled were Chinese, neglecting the racial differences.

## Conclusions

Our pilot study has showed that serum Rcn3 levels were significantly different between patients with IPF and CTD-ILD. Serum Rcn3 levels were significantly increased in patients with CTD-ILD and had a positive correlation with CTD-ILD severity presented by pulmonary function, but not in IPF. Furthermore, Serum Rcn3 levels might be a valuable biomarker to diagnose CTD-ILD.

## Data Availability

The datasets generated and/or analyzed during the current study are not publicly available due to the ownership of these data but are available from the corresponding author on reasonable request.

## Abbreviations

ILD: Interstitial lung disease
IPF: Idiopathic pulmonary fibrosis
CTD-ILD: Connective tissue disease-associated interstitial lung disease
UIP: Usual interstitial pneumonia
SP-D: Surfactant protein-D
Rcn3: Reticulocalbin 3
AECIIs: Type II alveolar epithelial cells
CRC: Colorectal cancer
TLC% pred: Predicted total lung capacity
FVC% pred: Predicted forced vital capacity
DLCO% pred: Predicted lung diffusing capacity for carbon monoxide
BALF: Bronchoalveolar lavage fluid
ROC: Receiver operating characteristic
AUC: Area under the curve
CI: Confidence interval
PPV: Positive predictive value
NPV: Negative predictive value
LRPT: Likelihood ratio of positive test
LRNT: Likelihood ratio of negative test
